# Creating supply chain resilience during and post-COVID-19 outbreak: the organizational ambidexterity perspective

**DOI:** 10.1007/s40622-022-00309-w

**Published:** 2022-04-27

**Authors:** Barbara Ocicka, Wioletta Mierzejewska, Jakub Brzeziński

**Affiliations:** 1 Institute of Corporate Finance and Investment, SGH Warsaw School of Economics, al. Niepodległości 162, 02-554 Warsaw, Poland; 2 Institute of Management, SGH Warsaw School of Economics, al. Niepodległości 162, 02-554 Warsaw, Poland; 3 Department of Logistics, Faculty of Management, University of Lodz, ul. Matejki 22/26, 90-237 Łódź, Poland

**Keywords:** Supply chain, Resilience, Ambidexterity, Crisis, COVID-19, L21

## Abstract

This study aims to investigate the significance of organizational ambidexterity (OA) in creating supply chain resilience (SCRES) during and after the COVID-19 pandemic. The methodological triangulation is applied in this study. A literature review, semi-structured online interviews and insights from open webinars serve as the sources of data. A framework, based on three pillars: validation, positioning and evaluation of business practices, is used for data analysis. The dependencies between OA activities and SCRES strategies are presented. The authors discuss their evolution during and in the post-pandemic period and outline the SCM trends in a strategic perspective. This paper investigates a pathway for closing the gap between OA theory and industry practice to create SCRES during and post-COVID-19 outbreak. This article starts the discussion on creating SCRES through OA. Future quantitative and qualitative research should explore the applicability of OA to enhance SCRES in a dynamic environment. Understanding the critical connection between exploitation and exploration practices and how OA influences SCRES provides valuable insight into the subject to supply chain managers supporting them in pursuing their roles successfully in the times of crisis. This study is focused on two concepts, OA and SCRES, of critical importance for how practitioners manage supply chains in the times of crisis. The resilience of supply chains to crises is crucial for the well-being of societies.

## Introduction

Increasing complexity and unpredictability of global business environment reveals itself not through gradual changes, but through periodic discontinuities. These changes are driven by technological and social shifts, economic and political conditions, current and potential competitors (Tushman and O’Reilly 1996), and nowadays by the COVID-19 outbreak. Today’s supply chains (SCs) as complex and global networks are vulnerable to those instabilities. Emerging disturbance causes problems along the SC and raises business risk (Lee and Rha 2016). Thus, building supply chain resilience (SCRES), that enables to cope with disruptions by quick and cost-effective reaction (Kochan and Nowicki 2018) gained considerable attention of both practitioners and researchers. SCRES permit to anticipate, adapt, respond and recover promptly from unpredictable events (Ali et al. 2017).

Recent studies have investigated the SCRES from various aspects (Aslam et al. 2020). Organizational ambidexterity (OA) is a new concept that gives original insights into SCRES. It refers to contradictory dualities such as efficiency (exploitation) and flexibility (exploration) (Severgnini et al. 2019). Some researchers indicate that OA becomes a paradigm for an organization facing complexity and uncertainty of the environment (Claudia and Mihaela 2019), required to sustain organizational success in a turbulent environment (O’Reilly and Tushman 2008), or even to ensure long-term survival in uncertain, volatile and rapidly evolving industries (Hansen et al. 2019). However, the understanding of the OA significance in creating SCRES remains limited. The research on this issue is relatively scarce (Aslam et al. 2020). Additionally, prior studies on SCRES are mainly theoretical (Ali et al. 2017) without overall assessment of practices under exploitation and exploration dimensions. Furthermore, only few studies recognize this issue in times of such strong turbulence as the COVID-19 pandemic caused (Ali et al. 2021; Ozdemir et al. 2022).

In this article, the ambidexterity lenses are implemented to provide better understanding of SCRES and extend knowledge by explaining the evolution of SCRES strategies and practices during and after the COVID-19 outbreak. Consequently, the aim of this study is to answer the following research questions:

- What is the significance of OA in creating SCRES during and after COVID-19 pandemic?
- How the SCRES practices evolve during and after COVID-19 pandemic?
- What are the supply chain management trends that may improve the SCRES under OA in the post-COVID-19 world?

To answer the research questions 47 practices pursued by companies operating during the COVID-19 outbreak were identified and evaluated in 25 semi-structured online interviews with professionals managing various business processes within international supply chains across different industries. The data analysis enabled to systemize and position selected practices according to the significance of two pillars of OA (exploitation and exploration) and explain its role in building SCRES strategies during and post-COVID-19 outbreak. We argue that ambidextrous activities (exploration and exploitation) are needed to implement reactive and build proactive SCRES strategies. OA enhance SCRES strategies not only during crisis but also in post-pandemic future.

This study advances SCRES literature in important ways. First, it adds OA perspective to discussion on creating resilient SC. The OA perspective in supply chain management (SCM) literature was under researched (Aslam et al. 2020). This study outline the significance of OA in creating SCRES during and after the COVID-19 pandemic by analysing set of practices. Explanation of OA role in SCRES building is important contribution to theoretical and practical discussion on preventing disruptions within supply chains. Next, this study explores SCRES strategies during crisis. Yet, only few studies consider SCRES strategies under crisis caused by COVID-19 pandemic (Ali et al. 2021; Ozdemir et al. 2022) that is a challenge different than before. The COVID-19 pandemic forced researchers and professionals to reconsider supply chain management (Ozdemir et al. 2022). This study contributes to the literature by giving insight into SCRES strategies and practices that were implemented during COVID-19 pandemic. Finally, this study implements dynamic analysis. It explains the evolution of SCRES strategies and practices during and post-crisis. It expands SCM knowledge by giving imperatives for managing future supply chains in the post-COVID-19 times.

The remainder of the article is organized as follows. It starts with the presentation of the SCRES definition and strategies. The next section introduces the idea of supply chain ambidexterity and explains its importance to supply chain resilience in the light of the literature review. Then comes the analysis of supply chain practices that demonstrates the use of exploitation and exploration activities in building SCRES strategies during and post-COVID-19 outbreak. Its culmination is the indication of the SCM trends in the post-COVID-19 world, which are discussed in the next section. The key conclusions and main research implications are outlined at the end.

## Literature review

### Supply chain resilience

The first wide-spread study on resilience in the context of the supply chain was launched in the early 2000s, following the disruptions to transport caused by fuel protests and the outbreak of Foot and Mouth Disease. After that, scientists from the Cranfield University dealing with the topic of resilience, stated that the subject requires thorough and in-depth research, as it is underestimated in literature and extremely important in business (Pettit et al. 2010). This research gap was filled by Christopher and Peck (2004), who developed an initial framework for supply chain resilience (SCRES), defining it as *the ability of the system to return to its original state or move to a new more desirable state after being disturbed*. This way of perceiving the SCRES features in numerous papers and can be described as the ability of a SC to withstand changes of steady-state and converge to the original state or to a new desirable state (Piers Ribero and Barbosa-Povoa 2018; Carvalho et al. 2012; Erol et al. 2010; Rice and Caniato 2003; Xiao et al. 2012). According to Hohenstein et al. (2015), SCRES is *the supply chain’s ability to be prepared for unexpected risk events, responding and recovering quickly to potential disruptions to return to its original situation or grow by moving to a new, more desirable state in order to increase customer service, market share and financial performance*. It is worth emphasizing that SCRES recognizes both the ability to absorb shocks in the form of extreme events and the adaptive capability to adjust to new circumstances (Brusset and Teller 2017) and is considered a responsive capability for a firm’s performance, as well as a key dimension of a firm’s survival. More recent definitions are more complex and complete, and they tend to combine several elements present in previous and simpler definitions (Piers Ribero and Barbosa-Povoa 2018). Kalamandi and Parast (2016) define SCRES as the adaptive capability of a supply chain to reduce the probability of facing sudden disturbances, resist the spread of disturbances by maintaining control over structures and functions, and recover and respond through immediate reactive plans to transcend the disturbance and restore the supply chain to a robust state of operations.

Although there are several various definitions of SCRES in the SCM literature originating from diverse disciplines, there is an overall multidisciplinary consensus as to the types of SCRES strategies. Most researchers and practitioners agree with their division along two main dimensions: proactive and reactive (Hohenstein et al. 2015; Dabhilkar et al. 2016; Tukamuhabwa et al. 2017; Cheng and Lu 2017). The distinction rests mostly on their role in building SCRES capabilities in different phases: pre-disruption, during disruption or post-disruption, generally taking into account whether they are employed proactively to avoid a threat or reactively to recover from it (Hendry et al. 2019). Ali et al. (2017), distinguish five core SCRES capabilities: to anticipate, adapt, response, recover and learn. Hohenstein et al. (2015) point out the four SCRES phases: readiness, response, recovery and growth. Wieteska (2019) highlights five SCRES abilities to anticipate, respond, recover, learn and improve. Tukamuhabwa et al. (2015) emphasize that certain strategies can be either proactive or reactive depending on when and why they are applied and, in addition, they indicate that some SCRES strategies are interrelated and reinforce each other.

The reactive approach is focused mainly on the ability to respond and recover (Sheffi and Rice 2005; Ponomarov and Holcomb 2009) or furthermore, to recover, learn and grow (Ali et al. 2017; Pertheban and Arokiasamy 2019) after a crisis takes place. It refers to building capabilities required to quickly recover from a disruption (Ponomarov and Holcomb 2009), ensuring access to resources necessary for recovery (Johnson et al. 2013) and creating capacities that can be used to cope with disruptions (Rice and Caniato 2003). Proactive elements of SCRES refer to SC resources that build and enhance the ability to anticipate disruption and achieve a disruption-avoidance status (Mwangola 2018). Ali et al. (2017) claim that proactive strategy is based on competences necessary to build capabilities in the pre-disruption phase, that ensure the readiness and the ability to anticipate threats. Hollnagel et al. (2006) point out that proactive resilience enables to recognize, anticipate and successfully defend against the risk before adverse consequences occur. According to Pertheban and Arokiasamy (2019), the proactive approach consists in taking action before final necessity occurs. It should be noted, that Hollnagel (2011) and Ali et al. (2017) propose a third type of SCRES strategy by adding so-called concurrent strategies to the former classification and defining them as rapid, initial responses during a disruption or in the immediate post-disruption phase. Due to their nature, concurrent strategies might be classified as reactive ones.

### Supply chain ambidexterity concept

The organizational ambidexterity (OA) is perceived as a phenomenon of tensions in ensuring company survival. It is defined as the ability of an organization to do two things simultaneously (O’Reilly and Tushman 2013), to pursue competing strategic orientations (Clauss et al. 2020) or to both explore and exploit (O’Reilly and Tushman 2013). OA integrates paradoxes into one complex construct (Claudia and Mihaela 2019) and strongly relies on the dynamic capability concept (O’Reilly and Tushman 2011; Yan et al. 2016; Popadiuk et al. 2018). An ambidextrous organization focuses on both “exploration of new possibilities” and “exploitation of old certainties” (March 1991). This concept is focused on exploration–exploitation trade-offs between units in one organization or between alliance partners (Aoki and Wilhelm 2017). Exploration and exploitation activities are perceived as contradictory but strongly interrelated (Wei et al. 2014) and reveal in companies' structures, behaviours and strategies (He and Wong 2004).

The ambidexterity concept is very rarely applied in SCM (Rojo et al. 2016). Kristal et al. (2010) define supply chain ambidexterity from the manufacturer’s perspective and present it as a manufacturer’s choice. It is conceptualized as a simultaneous pursuit of both explorative and exploitative SC practices. A manufacturer can exercise different exploitative and explorative practices with each partner within the SC subsystem. According to Aslam et al. (2020), SC ambidexterity is defined as *the ability to modify supply chain design to adapt according to the market changes while aligning the incentives of the supply chain partners.* Partanen et al. (2020) notice that supply chain ambidexterity depends on a manufacturer's efforts to refine and/or extend its existing resources and to develop new competencies. Aslam et al. (2018) argue that supply chains must seek to provide quick responses to short-term market changes and, at the same time, be able to adapt to resource base configuration to achieve long-term efficiency gains.

The ambidexterity concept can be implemented in SCM by developing practices, which help in both exploiting current competencies and exploring new ones. Generally, exploitation is focused on activities that help to transform resources into commercial ends (Pertusa-Ortega and Molina-Azorín 2018), improve existing operational processes (Blindenbach-Driessen and Van Den Ende 2014), components (Benner and Tushman 2002) and product-market domain (He and Wong 2004). According to March (1991), exploitation concerns refinement, efficiency, control and implementation (Blindenbach-Driessen and Van Den Ende 2014). It is related to exploiting existing strengths and using known solutions (Martin et al. 2019). Exploitation enables an organization to stay strong in actual activities (Blindenbach-Driessen and Van Den Ende 2014). Exploitation can be achieved by using the existing organizational resources and routinization (Aoki and Wilhelm 2017). Examples of exploitation activities applied to SC resources include supplier development, supplier qualification and automation of cross-organizational tasks (Rojo Gallego Burin et al. 2020). They are aimed at maintaining relationships with current suppliers, searching for SC solutions using the existing resources and leveraging current SC technologies (Lee and Rha 2016). Exploitation focusses on short-term benefits and measurable targets like cost reduction, reliability, risk reduction and the overall efficiency of the supply chain (Partanen et al. 2020). In the organizational learning approach, exploitation is related to the acquisition of knowledge by seeking, selection, processing of information and the betterment of existing routines through experience (Baum et al. 2000; Rojo Gallego Burin et al. 2020). Activities using the existing knowledge base are intended to refine current processes and technologies (Güemes-Castorena and Ruiz-Monroy 2020).

Exploration is the opposite of exploitation. Exploration allows to move quickly towards new opportunities (Birkinshaw and Gibson 2004), generate new possibilities (Blindenbach-Driessen and Van Den Ende 2014) and help to continuously renew and expand the knowledge base of an organization (Pertusa-Ortega and Molina-Azorín 2018). Activities related to it are associated with search, variation, experimentation and innovation (March 1991). Exploration enables an organization to change the direction and leap forward (Blindenbach-Driessen and Van Den Ende 2014). Exploration deals with the development of new SC competencies through experimentation and acquisition of new knowledge and resources (Kristal et al. 2010). In a supplier–buyer relationship exploration results in new routines (Aoki and Wilhelm 2017), enables environmental adaptability and leads to long-term success through learning and innovation (Partanen et al. 2020). Thus, it is focused on searching for SC solutions based on novel approaches and seeking creative ways to satisfy customers (Lee and Rha 2016). Examples of explorative practices within the supply chain include supplier innovation workshops and systems for cross-entity business intelligence (Rojo Gallego Burin et al. 2016).

OA can be implemented in a variety of ways. Most common approaches are: sequential ambidexterity, structural ambidexterity and contextual ambidexterity (O’Reilly and Tushman 2013; Ossenbrink et al. 2019). Sequential and structural approaches try to overcome generic conflict between exploration and exploitation through separation of those activities. In former approach, it is temporal separation and in latter approach structural separation (Tushman and O’Reilly 1996; Birkinshaw and Gibson 2004). In contextual approach, employees make choices between alignment-oriented and adaptation-oriented activities in their day-to-day work. That is some kind of temporal separation of activities, but emerging on individual level (Birkinshaw and Gibson 2004). Different studies proved that organizations apply in distinct configurations discussed approaches to ambidexterity (Fourné et al. 2019; O’Reilly and Tushman 2013). However, regardless of the approach, OA means implementing both exploration and exploitation practices.

OA concept can be very useful in SCM. Literature review provides evidence that properly implemented OA may increase both short-term and long-term competitiveness of an organization (Rosing and Zacher 2017), ensure its longer survival (Cottrell and Nault 2004), secure better financial performance (Derbyshire 2014), improve learning and innovation skills (Eriksson 2013), improve business model innovation (Minatogawa et al. 2020). Advantages of ambidexterity in the SCM concern value creation by using the relevant knowledge, partner satisfaction and access to resources, as well as improvement of business performance (Güemes-Castorena and Ruiz-Monroy 2020). Ambidextrous governance in SC has got a positive impact on innovation and cost performance (Blome et al. 2013) as well as on competitive advantage of a SC (Rojo et al. 2016), mitigates potential disruptions in the SC (Lee and Rha 2016), positively affects SC flexibility (Rojo Gallego Burin et al. 2016, 2020) and agility (Tuan 2016). Some researchers proved that ambidexterity enhance company’s resilience (Bechthold et al. 2021; Iborra et al. 2020; Stokes et al. 2019; Turner et al. 2018; Amah and Onwughalu 2017; Turner and Kutsch 2016) and positively impacts SCRES (Aslam et al. 2020). Eltantawy (2016) argues that SCRES capabilities are inextricably linked with the concept of ambidexterity. Ambidexterity allows building resilience to mitigate negative impact of SC disruption, maximize business performance, respond to market needs and adapt to the rapidly changing environment (Lee and Rha 2016). OA is perceived as an effective mechanism to achieve SCRES (Aslam et al. 2020), especially in a dynamic and uncertain environment. Previous studies proved that companies are able to survive and recover from external threatening by relying on ambidexterity capabilities (Iborra et al. 2020) which increase company's ability to allay and readjust to environmental disturbances (Bechthold et al. 2021). Thus, organizations that operate in turbulent and dynamic environment should adopt OA concept to remain resilient (Zhaxylyk 2020).

## Research methodology

**Fig. 1:**
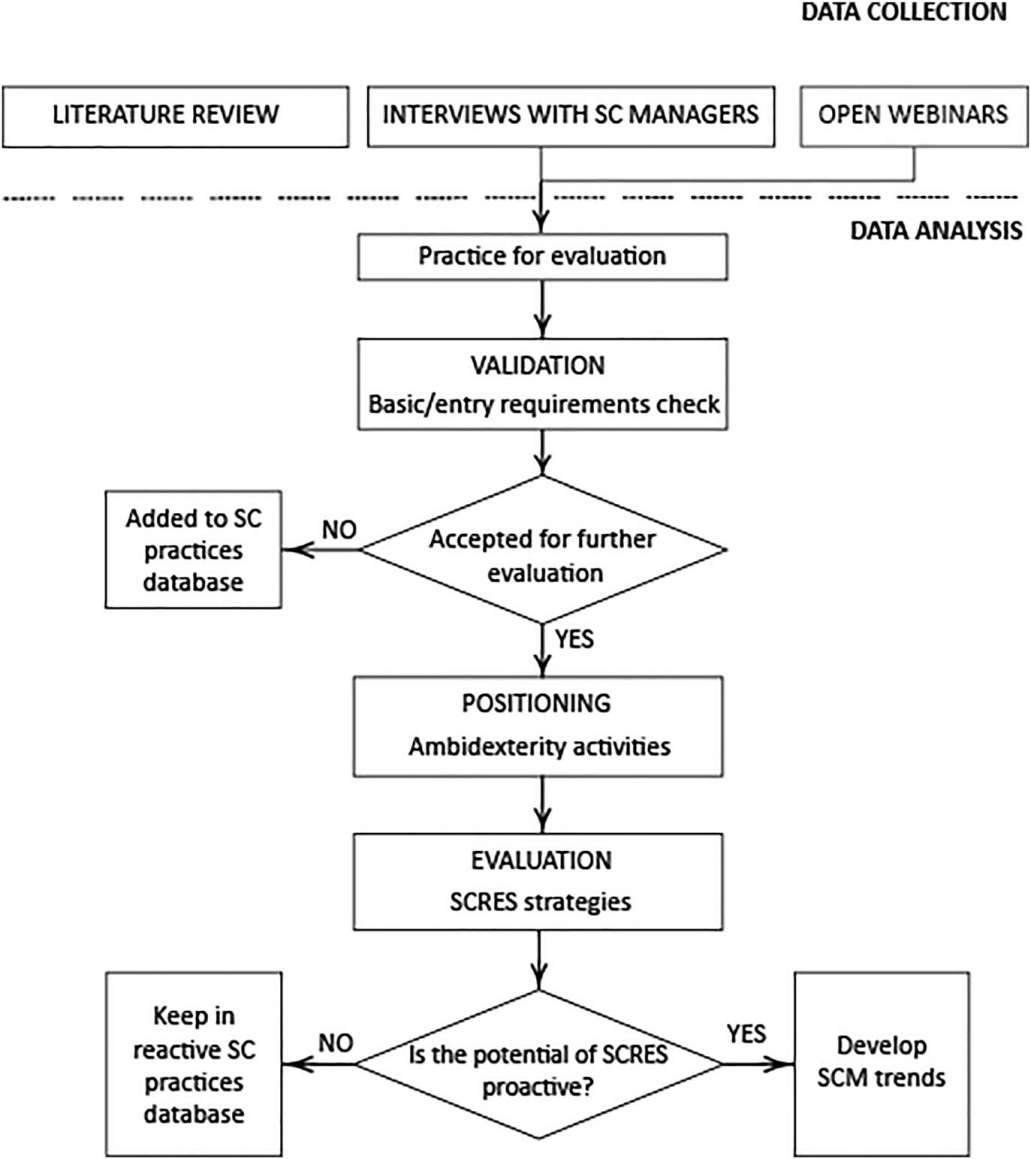
Research procedure. Source: authors’ own elaboration

Given that the objective of this article is to investigate the significance of organizational ambidexterity in creating supply chain resilience during and post the COVID-19 outbreak and to close the gap between theory and industry practice, the methodological triangulation can serve this approach. The entire research procedure is divided into two main phases: data collection and data analysis as presented in Fig. 1.

### Data collection

Three methods of secondary and primary data collection were used. Firstly, the authors conducted literature review to gain deeper insight into empirical data collection and analysis of the concepts of supply chain resilience, organizational ambidexterity and supply chain ambidexterity. The review covered articles selected based on the keyword searches (“supply chain resilience”, “organizational ambidexterity”, “supply chain ambidexterity”) in multiple research databases: Ebsco, Emerald, Science Direct, Web of Science, Scopus and Wiley. Secondly, qualitative research methods were used to identify business practices that have been implemented within SCM. Authors’ participation in open virtual webinars provided the opportunity to learn from SC managers and discuss with them the strategies and practices developed for the improvement of SCRES in times of the COVID-19 pandemic. The following webinars specifically helped to identify business practices:

1. *Procurement for sustainable growth. Procon/Polzak 2020* on 19–20 October 2020, http://konferencja-proconpolzak.pl/en/home-2019-en/,
2. *CSCMP CEE Supply Chain Conference* on 17 November 2020,
3. *European Economic Forum* on 3–4 December 2020, https://forum.lodzkie.pl/en/european-economic-forum-lodzkie2020/,
4. *Logistics networks reconfiguration during and post-COVID-19* on 19 December 2020.
5. *COVID-19, Supply Chain Resilience and Global Trade,* Center for Global Development Webinar on 4 December 2020, https://www.cgdev.org/event/covid-19-supply-chain-resilience-and-global-trade.

Furthermore, the authors carried out 25 semi-structured online interviews (app. 60 min each) with professionals managing various business processes within international supply chains across different industries. Main questions asked during the interview were the following:

1. What is the impact of the COVID-19 pandemic on your corporate strategy and business model? Is your company considering any changes to them in the post-pandemic period? If so, what changes do you envisage?
2. What changes has the pandemic caused to the structure of your company’s SC? Is your company planning SC reconfiguration after the COVID-19 pandemic? If so, to what extent?
3. What changes is the pandemic causing in business processes within your company’s SCM? Which of them have been developed during the pandemic and will be continued after it?
4. What is the importance of relationships with SC links in the current pandemic crisis? Is your company considering changes in relationships management for the post-pandemic new normal? If so, what are they?
5. What management tools (including technologies) support your company in SCM during the pandemic? Is your company planning a continuous development of some of them to address supply chain challenges after the COVID-19 outbreak? If so, what are they?

The interviews were held between 6 March and 28 June 2020. The expert interviews collected during the research study were transcribed and pre-processed for coding purposes. Appendix 1 includes a dataset containing the respondent's designation along with quotes of statements directly relevant to the focus of the study. Quoted extracts from the interviews were transcribed and coded into unified and numbered SC practices. The structured data were subjected to a deeper analysis.

### Data analysis

The empirical qualitative data analysis was conducted using three stages of assessment methodology: validation (1), positioning (2) and evaluation (3). As a first step, basic, entry requirements check was carried out. The authors checked whether the identified practice was caused by the pandemic pressure. Only pandemic-induced practices were accepted for research data analysis. At parallel, practices have been cleaned up by excluding duplicates. As a second step, the selected practices were systemized and positioned according to the significance of two pillars of OA, which are exploitation and exploration. The third stage was devoted to the evaluation of practices in the light of SCRES strategies. The authors evaluated each practice taking into account types of SCRES strategies and their evolution during and post-COVID-19 outbreak. Furthermore, based on a dynamic approach, the authors explored the future potential of practices in the light of SCRES strategies and deeper discussed significant trends for SCM in the post-COVID-19 world.

## Research results

### SCM practices during the COVID-19 outbreak

The in-depth analysis of qualitative empirical data in line with the above-mentioned research procedure allowed to identify 47 practices pursued by companies operating during the COVID-19 outbreak. As a result of their validation, the list was created as presented in Table 1. At the next positioning stage, particular practices were classified as exploitation or exploration ambidexterity activities within SCM. The first type is related to the exploitation of resources already owned by companies and use of existing strengths and known solutions. During a pandemic, these practices are focused on maintaining continuity of operations by leveraging resources available beforehand. The second group consists of exploration practices aiming at creatively finding new solutions and taking on emerging opportunities.

**Table 1:** Validation, positioning and evaluation of SCM practices during COVID-19 outbreak

	Validation	Positioning	Evaluation
No	SCM practices	Ambidexterity activities	SCRES strategy^a^
1	Changing the criteria for selecting suppliers	Exploitation	R
2	Suspension and recalculation of contracts ready to be signed		R
3	Increasing inventory levels in local warehouses		R
4	Increasing inventory levels in supplier locations		R
5	Consolidate deliveries from multiple vendors		R
6	Extending order fulfilment dates		R
7	Supplier base mapping and monitoring		R
8	Risk management related to the supplier base		P
9	Changes and control in managing purchasing budgets		C
10	Production shutdown		R
11	Maintaining the continuity of production processes		R
12	Increasing the frequency of deliveries		C
13	Checking the level of inventory in SC intermediary links		C
14	Increasing inventory of finished products on SC demand side		R
15	Assortment rationalization		C
16	Changes in the product offer oriented to waste reduction		P
17	Transition of employees to the remote work model		R
18	Setting priorities in managing business processes in the SC		C
19	Increasing the salaries of employees		C
20	Lowering the salaries of employees		C
21	Changing the forms of employment to flexible ones		R
22	Know-how exchange between partners in the SC		P
23	Global sourcing	Exploration	P
24	Increasing the importance of local suppliers in isolated regions		R
25	Searching for sources of supply for new purchase categories		R
26	Development of multi-channel purchasing strategies		R
27	Establishing contacts with suppliers using online platforms		R
28	Digitalization and automatization of purchasing processes		R
29	Multiple sourcing		P
30	Reconfiguration of purchasing processes		C
31	Supplier relocation		R
32	Introducing new product categories to the production assortment		R
33	Domestic production		R
34	Decentralization of distribution centres		R
35	Changes in inventory management		R
36	Developing multi-channel distribution		R
37	Introducing new product categories to the commercial assortment		R
38	Introducing new product categories as sources of income		R
39	Developing e-commerce distribution		R
40	Developing relationships with customers using online platforms		R
41	Reconfiguration of customer order fulfilment process		R
42	Starting e-business		R
43	Reformulating goals in SCM		R
44	Using of new technologies in business process management		R
45	Logistics processes automation		R
46	Prediction and application of artificial intelligence possibilities		P
47	Logistics processes reconfiguration		C

Source: authors’ own elaboration

^a^*P* proactive, *R* reactive, *C* concurrent

The next step was the evaluation of the practices against SCRES strategy with reference to its three types: concurrent (C), reactive (R) and proactive (P). Reactive practices are, by definition, responses to disruptions caused, in this case, by a pandemic. By nature, these actions are intended to allow the organization to address issues as they arise, but also to learn lessons about further applicability after a disruption has occurred. Concurrent practices are similar in nature to reactive, but are distinguished by their abruptness of application due to the unpreparedness of companies for an unforeseen COVID-19 outbreak. Proactive practices refer to activities that build and enhance the ability to anticipate disruptions and achieve successfully defend against the risk before adverse consequences occur.

Most of the practices carried out at the upstream supply chain, related to purchasing, sourcing and supplier relationship management, were reactive in their nature. The need for changes in business processes such as raw materials acquisition and components production was strongly emphasized. Closing borders and reducing air connections made it necessary to search for suppliers on local markets and increase importance of local suppliers in isolated regions and thus minimizing foreign supplies. Nearshoring was a frequent remedial practice when the local market was unable to meet supply chain expectations. Companies, heavily dependent on supplies from the Far East markets, began looking for business partners close to the borders of their home countries. Changing where goods are acquired is one side of the coin. On the other hand, the typical reaction was to control inventory levels in companies’ facilities and at suppliers' locations. All SCs links made efforts to increase inventory levels for raw materials, components, modules, systems and semi-finished products. Difficulties with access to direct production supplies and communication barriers have accelerated the development of activities towards consolidation of deliveries originating from multiple vendors. The criteria for selecting suppliers have therefore also changed. In the conditions of crisis, the role of cost criteria has decreased. Instead, the continuity of supply and flexible payment deadlines began to be guided. Managers also emphasized the importance of inventory levels and business confidence, that are closely related to the development of supplier relationships. SRM is though another very important area within SCM under conditions of uncertainty. Especially trust between trading partners has in many cases been put to the test of economic collapse. Trade was disrupted by factors such as shortening payment terms and renegotiating contracts ready to be signed. Decisions about whom to sell the strategic goods began to be made based on the existing relationships and the quality of the so far cooperation. Another activity typical of the pandemic response was establishing and developing communication through online platforms. This technological trend can also be observed in the case of digital transformation of purchasing processes and increasing control over purchasing budgets.

The most far-reaching solution taken by companies was to shut down production. There were several reasons for such a categorical decision—reduced demand, lack of raw materials, willingness to limit inventory of finished products. At the same time, other companies focused efforts on maintaining the production continuity in cooperation with purchasing departments—so as to guarantee the necessary resources for production lines. Maintaining production was in many cases associated with a complete reorientation of this process, abandoning production abroad and starting domestic production. The factor contributing to such a change was the rationalization of the assortment and the introduction of new product categories at the expense of stopping production of goods for which demand has drastically decreased.

Distribution is the SC process, where the changes caused by the pandemic have been most visible to consumers. Some of them were the result of the changes in production described above. New product categories appeared on the market, some products were withdrawn from sale, to sum up the assortment was rationalized. However, the key change concerned the methods of selling and delivering products. Companies, that have not done this so far, have started to develop multi-channel distribution. The e-commerce market has grown drastically with the accompanying services. Due to the care for customers and employees, simplified forms of contactless deliveries have been developed, and formalities have been reduced to a minimum. The care for the client also manifested itself through the practices related to its remote service. Communication and relationships with clients have been developed via digital platforms. To satisfy their psychological needs related to contact with other people, companies developed not only systems of chats and sales forums, but allowed them to return to telephone contact, which was losing its importance. Major changes also took place in the area of inventory management of finished products and their flows. Distributors began to monitor their suppliers' inventory levels to a greater extent while maximizing their own levels. In many cases, it was decided to decentralize distribution centres and change the way of managing in order to be able to flexibly respond to the increased demand for e-commerce services.

The results of the conducted research allowed to identify a small number of practices that were proactively implemented during a pandemic. This is due to the sudden COVID-19 outbreak and the focus of companies on responding to this threat. Proactive actions, that companies were taking during this time, mainly relate to the sourcing area. A very important factor from the perspective of business continuity is the diversification of supplier base. The multiple sourcing ensures a security in the case of discontinuation of supplies from one of the sources, as well as the possibility of dividing the volume of orders into multiple suppliers. It is related to another practice – global sourcing, aimed at expanding supplier base in a geographical scope.

### Evolution of SCM practices and trends post-COVID-19 outbreak

**Fig. 2:**
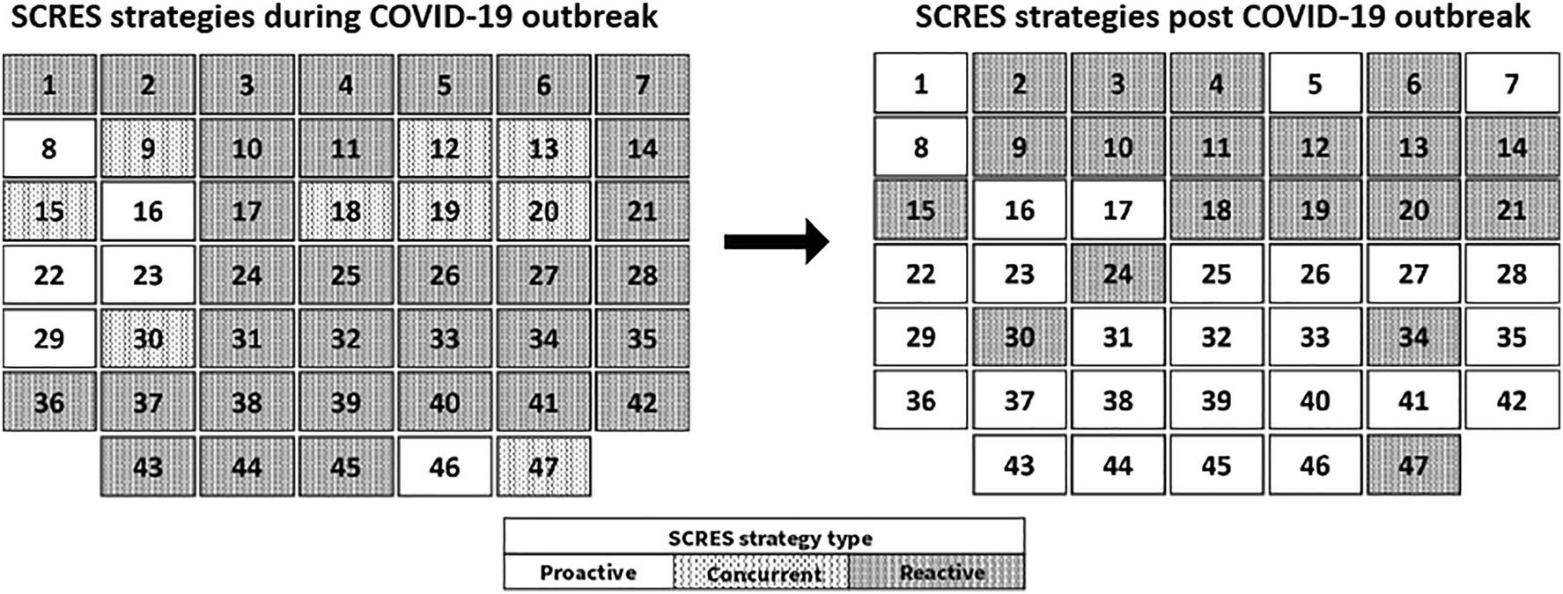
Evolution of practices in the light of SCRES strategies in the post-COVID-19 stage. Source: authors’ own elaboration

The added value of the assignments made at the evaluation stage is demonstration of a dynamic change of SCRES strategy. The practices were assessed not only in terms of their application during the COVID-19 outbreak. Based on the advanced analysis of the qualitative data, it was also possible to evaluate their character in the post-COVID-19 future. Figure 2 shows the evolution of practices in the light of SCRES strategies over time. At this point, it is worth noting that the practices assessed as “concurrent” during the COVID-19 outbreak have been included in the reactive ones. This was supported by the fact that, in essence, these actions were a quick “ad hoc” response to emerging challenges. Thus, by their nature they correspond to reactive actions. The catalogue of good practices for managers in the post-pandemic era refers to strategies that will be proactive in post-pandemic times and covers those that will remain or became reactive. On the one hand, some ad-hoc solutions are so economically ineffective that they will remain in the "just as needed" reactive sphere (e.g., production shutdown). On the other hand, some of the solutions, such as remote work, will remain proactive ones in the future, as they are effective in the opinion of managers. Finally, a large number of identified solutions have a significant potential to evolve from reactive to proactive SCRES strategies in the post-COVID-19 outbreak phase.

**Fig. 3:**
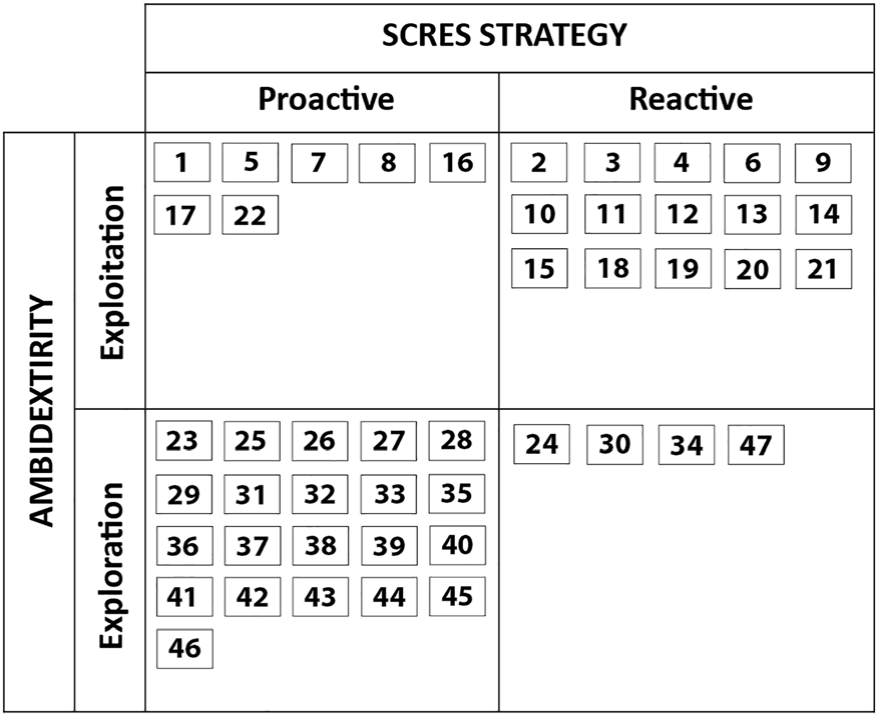
SCRES practices in the organizational ambidexterity perspective in the post-COVID-19 outbreak phase. Source: authors’ own elaboration

Despite the evolution of practices in relation to the SCRES strategy over time, their nature does not change in terms of ambidexterity activities. They were arranged in relation to both discussed issues of OA and categorized referring to the strengthening or building the resilience of the future SC (Fig. 3).

The SCRES strategies under organizational ambidexterity were classified into four categories:

- *Reactive SCRES strategy based on exploitation* This strategy includes a set of practices and potential actions of managers in the event of disruptions to keep business continuity. It was implemented during the COVID-19 outbreak and its nature in the future can be described as “only when needed”. It concerns activities based on internal SC resources that can be implemented relatively quickly. Regarding SCRES, reactive practices can be linked to the respond and recover phases.
- *Reactive SCRES strategy based on exploration *The essence of this strategy is taking corrective actions in response to disruptions, based on solutions that go beyond the resources of the SC. Most pre-pandemic practices that were “reactive exploration” have evolved towards proactive solutions.
- *Proactive SCRES strategy based on exploitation* This strategy includes practices aimed at systematic strengthening of the SCRES, based on the exploitation of available resources. They are typical of the earlier indicated SCRES readiness and growth phases. Organizations need to adopt a proactive approach to ensure resilience needed to absorb and avoid potential disruptions by not only returning to the original state through adaptation, but by surpassing it through developing specific elements to boost performance.
- *Proactive SCRES strategy based on exploration* This category includes practices aimed at increasing the SCRES by using external resources and searching for new forms of securing business operations and innovations. By incorporating these activities into proactive strategy, managers adopt a forward-looking approach to threats and develop an anticipatory capacity of enterprises.

There are two types of proactive post-COVID-19 practices. The first one is mainly based on the exploitation of available resources. The second category includes practices aimed at increasing the SCRES by using exploration capabilities to search new external resources and forms of managing business operations. We classified all proactive practices oriented both to exploitation and exploration into four groups as presented in Table 2 to reflect main trends in proactive SCRES strategies development.

**Table 2:** Proactive SCRES practices vs SCM trends in the post-COVID-19 world

SCM practices		SCM trends	
Introducing new product categories to the production assortment Searching for sources of supply for new purchase categories Changes in the product offer oriented to waste reduction Introducing new product categories to the commercial assortment Introducing new product categories as sources of income		Products assortment rationalization and development	
Reformulating goals in SCM Risk management related to the supplier base Supplier base mapping and monitoring Consolidate deliveries from multiple vendors Starting e-business Developing multi-channel distribution Know-how exchange between partners in the SC Transition of employees to the remote work model		Reorientation of supply chain management strategies	
Global sourcing Development of multi-channel purchasing strategies Multiple sourcing Supplier relocation Domestic production Development of e-commerce distribution Reconfiguration of customer order fulfilment process Changes in inventory management			Reconfiguration of supply chain structures, processes and relations
Using of new technologies in business process management Logistics processes automation Establishing contacts with suppliers using online platforms Digitalization and automation of purchasing processes Developing relationships with customers using online platforms Prediction and application of artificial intelligence possibilities			Technological transformation of business processes and relationships management

Source: authors’ own elaboration

The identified trends are as follows: product assortment rationalization and development, reorientation of SCM strategies, reconfiguration of SC structures, processes and relations, as well as technological transformation of business processes and relationships management, and will be discussed in the next section. In the face of changing economic conditions, companies explore the business environment thus preparing themselves for market uncertainty. By incorporating these activities into proactive strategy, managers adopt a forward-looking approach to threats. Pandemic-induced practices will evolve in many cases to strengthen resilience and acquire anticipatory capacity in the post-pandemic COVID-19 outbreak phase.

## Discussion

Researchers characterize the significance of the COVID-19 pandemic for supply chains management as a devastating impact (Chowdhury et al. 2021), an extraordinary disruption (Ivanov 2020a, b), an unprecedented humanitarian crisis (Farooq et al. 2021). SCRES strategies in the light of pandemic belong to four broad themes in publications and attention of researchers is focused on the three SCRES dimensions: preparedness, response and recovery (Chowdhury et al. 2021). Research findings presented in this article confirm that reactive SCRES strategies dominate during the COVID-19 outbreak. However, experts from academia and business practice agree that the future SCM strategic objectives should better reflect elements and capabilities of SCRES (Hong and Kochar 2020; Linton and Vakil 2020; Steinberg 2020). In the highly uncertain post-COVID-19 business environment, it’s very important to build proactive SCRES. The evolution of different types of SCM practices applied during the COVID-19 outbreak to proactive SCRES strategies is a significant chance for growth. Following such a scenario and analysing proactive potential of identified practices, we propose imperatives for managing future supply chains in the “new normal” post-COVID-19 business environment.

*Product assortment rationalization and development* The research findings shed light on trends in product strategies emerged during the pandemic. The first trend is the product range rationalization. As the survey results outlined, companies exploited all possibilities to make changes in the product offer to reduce and eliminate waste. As Shih (2020) emphasizes, managers revisited the trade-off between product variety and capacity flexibility. The difficult and demanding post-pandemic market competition will strengthen this need. The second trend is product assortment development, reflected in demand increase on an unprecedented scale or exploration practices of searching for sources of new purchasing categories, introducing new product categories to the production and commercial assortment structures. According to Kang et al. (2020), product line extension is a desirable marketing strategy to satisfy urgent needs in the pandemic times and generate additional revenues. There are many examples of SCs development in the pharmaceutical industry, including medical supplies (e.g. thermal scanners, essential drugs, sanitizers, ventilators and protective face masks) and equipment (e.g. for checking, testing and monitoring). The most important case is designing and managing a completely new global and complex supply chain of coronavirus vaccines. New product categories have appeared especially in high-tech sectors. The IT sector has developed new products and services, enabling and supporting remote work of employees, as well as technologies for automation, robotization and digitalization of business processes. All of the above-mentioned sectors will remain strategic in economies and companies representing them plan to invest in innovations as well as to explore market chances in the post-pandemic phase.

*Reorientation of SCM strategies* The most important managerial imperative is to reshape strategies and reformulate strategic goals of SCM in the post-COVID-19 business environment. Generally, the COVID-19 crisis provides SC stakeholders with an opportunity to ask critical questions and rethink fundamental aspects regarding “hyper-globalization” (Madhok 2021). According to Shih (2020), product and logistics strategies need to be reassessed and reviewed, starting by mapping the full extent of supply networks, identifying vulnerabilities, and furthermore, uncovering and addressing the hidden risks. The importance of risk and crisis management as pillars for building SCRES strategies will increase significantly in a post-pandemic world. Viable SC models are especially gaining attention in the literature because of such main characteristics as stability, robustness, resilience and viability (Ivanov 2020a). Based on the empirical data analysis, we verified that companies have applied SCRES activities during the COVID-19 outbreak and will develop them after the pandemic crisis through both exploitation and exploration capabilities. Ambidextrous activities will be continuously used and improved in the post-pandemic future for implementing reactive and building proactive SCRES strategies. This finding is an important lesson learned for SCM in times of crisis. The main assumption is that companies need OA to enhance SCRES in the strategic perspective.

*Reconfiguration of SC structures, processes and relations* SC reconfiguration will gain a strategic importance as risk management strategy and reconfiguration projects will follow revised SCM strategies in the post-COVID-19 world. Companies need to adapt their SC designs in response to global pandemic COVID-19 and in the face of new future challenges. The results of McKinsey surveys proved the significance of the need for SC reconfiguration, 73% of respondents encountered problems in the supplier footprint and 75% of them faced issues in the production and distribution footprint that require changes in the future (Alicke et al. 2020a, b). However, there is no uniform opinion among experts and researchers regarding the direction of supply chain reconfigurations. The need for the end-to-end de-risk SCM is much easier to implement in regional and local structures. During the pandemic, the role of local and regional supply chain links and their location has increased significantly, highlighting the importance of decentralized structures for resources availability and processes flexibility. Moreover, companies made a huge effort to eliminate single-source dependencies and diversify sources on the supply side. On the one hand, the development of practices such as domestic production, nearshoring or even local sourcing reveals the general deglobalization and regionalization of trends. On the other hand, however, based on the research findings, global sourcing has remained a proactive SCRES strategy oriented at the exploration of new sourcing markets and suppliers. New criteria of suppliers selection will emerge, like, e.g. recovery time, which means how quickly are they able to recover from a disruption. As experts argue, the proactive development of global sourcing and geographical diversification trends is *the obvious way to address heavy dependence on (…) a single factory, supplier or region is to add more sources in locations not vulnerable to the same risks* (Shih 2020). Van Hoek (2020) listed the need to balance global sourcing with nearshore and local sourcing among the key levers for de-risking supply chain in the post-COVID-19 world. Therefore, it should be considered that the reconfiguration projects will be hybrid by their nature. Aylor et al. (2020) identified three the following models: revised, migrated and regionalized global supply chains, emphasizing that the starting point and the speed of change will vary significantly across industries. The UNCTAD published the expertise according to which the COVID-19 pandemic will reinforce relocation, reshoring, diversification and regionalization within the SCM (Fortunato 2020). The reconfiguration of logistics processes based on new revised logistics strategies deserves special attention. The dynamic changes in transport, warehousing and inventory management will be proactively developed to provide greater reliability and enhance SC competitiveness. One more characteristic of SC reconfiguration projects during the pandemic is the creation of multi-channel structures in distribution. Market leaders will proactively explore innovations in multi-channel and even omni-channel business processes management facing such challenges as disrupted demand patterns and an explosion of consumer request for online shopping service (Schleper et al. 2021). To conclude, supply chain reconfiguration will gain strategic importance in the post-pandemic era as one of the strategies for risk avoidance and resilience reinforcement and companies will use exploitation and exploration dimensions of supply chain ambidexterity for its successful implementation.

*Technological transformation of business processes and relationship management* Technological transformation of business processes has had one of the greatest impacts on SCM in the COVID-19 pandemic. As Frederico (2021) proves, the Industry 4.0 technologies might play a crucial role for SC responsiveness and resilience to future disruption events. Both exploitation and exploration of advanced technologies such as Big Data, Blockchain, Artificial Intelligence, 3D printing, robots, cyber-physical-biotech or cognitive systems can help in developing SCRES. Researchers and practitioners discuss which technologies should be mostly exploited and which have the greatest potential to be explored in the future, moreover there are research questions focused on new ways in which they may contribute to the development of models, frameworks, policies and applications to create a safer post-COVID-19 era (Barnes 2020). KPMG (2020) reported that digital transformation will enhance SCRES in the new reality after COVID-19, directing the attention to advanced track and trace technologies, blockchain, predictive analytics and cognitive decision centres. Considering a similar path, Baker McKenzie (2020) indicates both human intelligence and data collection as assets critical to the identification of potential vulnerabilities and the creation of SCM strategies to minimize, manage or eliminate risks. Professional experts point out to the potential of the 3D printing technology development, exploited to produce face masks and shields, bespoke ventilator parts or hands-free door openers, that offers huge opportunities of exploration for just-in-time more localized production (Schell 2020). Ivanov and Dolgui (2020) highlight the importance of a digital SC twin for ensuring end-to-end visibility and business continuity. Based on the research outcomes, we argue that the development of AI applications has a critical importance for exploration of SCRES strategies based on prediction and anticipation of crisis changes. Generally, the aftermath is progressive automation, robotization and digitalization of business processes and relationships management. The achievement will be a higher level of the implementation of the Industry 4.0 technologies within supply chain management, which certainly be increasingly advanced and cognitive in the Industry 5.0. To sum up, companies will need organizational ambidexterity – on the one hand, exploiting the today’s and on the other hand, exploring the future potential of technologies.

## Conclusions, implications and limitations

Today’s organizations need to be ambidextrous to achieve long-term success or even to survive in a rapidly changing environment (Bahar and Akhtar 2020). Dynamic environment necessitates balancing between exploitation and exploration in order to be effective (Birkinshaw and Gibson 2004; O’Reilly and Tushman 2004; Raisch et al. 2009). Some researchers argue that properly pursued OA will enhance organizational performance (Simsek 2009; Kassotaki et al. 2019) and SCRES (Lee and Rha 2016; Aslam et al. 2020). The latter is crucial in the times of crisis for economic and social reasons. During uncertain times companies should focus on activities that enable exploiting current competencies and exploring new ones. In our opinion, an ambidextrous supply chain resilience might be defined as *the ability of the supply chain to achieve SCRES capabilities through exploitation and exploration practices*.

The results of our research study confirm high importance of OA in creating SCRES during and after COVID-19 pandemic. Both exploitation and exploration practices within SCM have created a basis of SCRES strategies developed during COVID-19 pandemic. Furthermore, identified practices evolve in terms of proactive and reactive nature of SCRES strategy. Most of them will become proactive ones, based on exploitation or exploration, after the COVID-19 pandemic. The in-depth analysis of proactive practices revealed SCM trends, that may improve the SCRES under OA in the post-COVID-19 world, namely such as: product assortment rationalization and development, reorientation of SCM strategies, reconfiguration of SC structures, processes and relations, technological transformation of business processes and relationship management.

The study contributes to the literature on SCM and SCRES in important ways. First the research extends understanding of SCRES by explaining the evolution of SCRES strategies and practices during and post-crisis based on the study of global COVID-19 pandemic. Second, it extends current research on the role of OA within supply chain management, highlighting issues related to SCRES strategies. Third, conclusions make a contribution to the research agenda focused on managing supply chains in the times of crisis. There is relatively little research linking SCRES with OA concept in the crisis perspective.

Future research may address the following problems:

1. How to create and implement SCRES strategies, using exploitation and exploration activities?
2. How to develop synergies between exploitation and exploration practices in the light of the OA concept to enhance SCRES?

There are several practical implications of the study for SCM. First, the organizational ambidexterity concept allows companies to survive and evolve through mitigation and overcoming disruption within SCM. This finding suggests that supply chain managers must develop new competences including exploitation and exploration capabilities for building SCRES. Second, the evolution of SCRES strategies based on ambidextrous activities informs managers that crisis may be a chance for proactive change and creation. Our research findings show that companies use both exploitation and exploration business practices during the COVID-19 pandemic. Organizational ambidexterity has proven to be a key ability in managing business processes in supply chains. Moreover, exploitation and exploration activities were used in the implementation of all types of SCRES strategies. It is worth emphasizing that the approach should be dynamic and focused on the evolution of practices depending on their development potential after the COVID-19 pandemic. Due to the managerial need to develop mainly proactive SCRES strategies, the authors outlined the SCM trends.

This article starts the discussion on creating SCRES through OA. Although the presented qualitative study contributes to SCM knowledge and managerial practice, the need remains to understand interdependencies between exploitation and exploration activities in more details. Future quantitative and qualitative research should explore the influence of OA on SCRES and its applicability to successfully enhance SCRES in the “new normal” business environment. It is desirable to conduct an international research, that can provide valuable insights.

## Appendix 1 Data set after coding

**Table:** 

Respond	Quotation	SC practice	Practice code
RES-1	Moving purchasing activity to the Internet can be a major challenge for companies that have so far preferred face-to-face contact. It involves the need to use remote communication platforms to maintain relationships with business partners. This applies to both customers and trading partners on the supply side.	Developing relationships with customers using online platforms	40
		Establishing contacts with suppliers using online platforms	27
	In addition to launching their online activities, companies should also think about preparing their employees properly. Investing in the development of employees' digital skills is essential, as working from home could become a daily reality for many professions even after the end of the pandemic.	Starting e-business	42
		Transition of employees to the remote work model	17
RES-2	Our factories recorded a 42% drop in turnover compared to March last year. Due to a decrease in demand for the manufactured products, the company's management decided to stop production in the factories. The aim of our company, which employs around 3,500 people, was to reduce costs in order to be able to cover fixed costs, such as salaries for employees.	Production shutdown	10
RES-3	In order to minimize the risk of the drug not being available in one of the countries, the company has significantly increased the stock levels in its local storage areas.	Increasing inventory levels in local warehouses	3
	Sales and sourcing staff had to meet the supply requirements of their supply chain department. They looked for as many global contractors as possible who could supply components used in clinical trials such as packaging, labels and sources of commercial drugs to support experimental therapies.	Global sourcing	23
	The increase in the amount of medicine in local warehouses was significantly increasing the monthly storage costs. It was therefore important to find local manufacturers in isolated countries who could supply commercially manufactured equipment per need.	Increasing the importance of local suppliers in isolated regions	24
RES-4	Our company sells through two channels: in-store pharmacies and online sales. With the development of the pandemic, it began to develop product categories that were previously not in demand. The categories such antibacterial gels, gloves, masks, visors were until now practically a forgotten assortment. There was no demand for these products. This changed practically overnight. The category buyer had to research the market in depth and really build an offer for the consumer from scratch. At the present time, this product category shows potential to catch up in sales. If not for the pandemic this category would definitely not have become the leading and mainly sales generating category. Once the pandemic is over, the category is still to be maintained, as it is assumed that people will become more aware of taking care of their safety and will develop the habits of disinfecting, using gloves and masks in everyday life.	Introducing new product categories to the production assortment	32
		Introducing new product categories to the commercial assortment	37
		Searching for sources of supply for new purchase categories	25
		Introducing new product categories as sources of income	38
RES-5	Our supplier was asked to increase the rotating buffer stock in the warehouse. Any delays due to extended border controls did not affect deliveries directly to production, so we ruled out the risk of a shortage of raw material.	Increasing inventory levels in supplier locations	4
RES-6	We received information from suppliers about difficulties caused by staffing in their companies. They asked us to combine deliveries and warned of extended lead times. We have also definitely minimized overseas deliveries.	Consolidate deliveries from multiple vendors	5
		Extending order fulfilment dates	6
		Changing the criteria for selecting suppliers	1
RES-7	As shopping during the pandemic was made significantly more difficult for consumers, we decided to expand our business to include online sales, introducing new technological tools.	Developing multi-channel distribution	36
		Starting e-business	42
RES-8	I mean that the coronavirus pandemic is forcing companies to change the way they do business and, therefore, to buy services they have not practised before. Our example shows that companies are choosing to cooperate and purchase new technologies.	Using of new technologies in business process management	44
RES-9	Purchasing leaders were challenged to make decisions that would allow the company to weather this period least severely. The first task for the purchasing departments was to cut costs sharply, as purchasing and the organization of the supply chain were responsible for around 50%-80% of costs in the organization. It was important to guarantee liquidity and financial stability in view of rising transport prices and increased production costs.	Reformulating goals in SCM	43
		Setting priorities in managing business processes in the SC	18
	An equally significant challenge was the assessment of existing risks among key suppliers. Recognizing the likelihood of unexpected implications allowed purchasing, production and sales priorities to be set correctly.	Risk management related to the supplier base	8
	We decided to implement a Source-To-Pay (S2P) purchasing process combined with Budget-To-Pay (B2P). This solution made it possible to make only purchase decisions whose repayment is certain.	Changes and control in managing purchasing budgets	9
		Using of new technologies in business process management	44
RES-10	Since the beginning of the pandemic, the managing director has taken precautionary measures to stockpile adequate supplies of products in case the need arises to isolate workers or quarantine them for a minimum of 2 weeks.	Increasing inventory levels in local warehouses	3
		Maintaining the continuity of production processes	11
	When I was informed about the progress of negotiations, I noticed that suppliers, despite the purchasing history on our side, worried about the situation and the further development of the pandemic, demanded shorter payment terms, despite waiting longer than usual for delivery. In many cases, companies even asked for prepayments, explaining their fear of the impact of the pandemic.	Suspension and recalculation of contracts ready to be signed	2
	Having once again verified the situation on the market, the management took new steps to increase control over purchases made by the company. This was primarily related to budget restrictions and the renewed verification of the purchasing needs of individual organizational units of the company.	Reformulating goals in SCM	43
		Changes and control in managing purchasing budgets	9
RES-11	Reflecting on the steps taken in the company where I work, I notice that all companies in this sector have reacted in exactly the same way—by increasing orders for raw materials/materials to increase stocks and by increasing cover on finished goods.	Increasing inventory levels in local warehouses	3
	We have started to use increasingly sophisticated technologies to manage purchasing and the entire supply chain. These are intended to give some security and stability to the business. COVID-19 has in a way forced our investment in new technology and digitalization.	Digitalization and automatization of purchasing processes	28
RES-12	We used “dispensing machines”—intelligent machines that connect to each other, analyse purchases, consumption reports and order them themselves. The role of the buyer is simply to draw up a contract with the suppliers.	Digitalization and automatization of purchasing processes	28
RES-13	The pandemic has largely driven companies to automate warehouse processes, transport and other repetitive activities that can be entrusted to robots. We use these solutions, for example, in managing warehouse operations, optimizing transport, customer deliveries or value-adding services. With the support of augmented reality, it was possible to complete 9,000 orders covering 20,000 items within a certain timeframe, thus confirming faster execution and error-free operations.	Logistics processes automation	45
RES-14	Tools that support strategic decision-making under uncertainty are becoming increasingly important. The supply chain intelligence system, which is based on artificial intelligence, is a programme that facilitates company management, including purchasing. It makes it possible, among other things, to identify any problems and estimate their possible consequences even before they occur, and on the basis of previous experience it facilitates decision-making, in time even allowing for their certain automation.	Prediction and application of artificial intelligence possibilities	46
RES-15	Monitoring and mapping suppliers, has become key in purchasing management, as there is no way to run a globally dispersed supply chain without knowing the daily news that may cause disruption in the days ahead. In terms of supply source diversification, we need to reduce dependence on a single supplier to introduce a multi-sourcing system. This is a good safeguard in case of unplanned deliveries, we have the ability to split the volume of orders across multiple suppliers.	Supplier base mapping and monitoring	7
		Multiple sourcing	29
		Development of multi-channel purchasing strategies	26
	In addition, more precise inventory strategies will be developed to safeguard the business against supply disruptions in supply chains.	Changes in inventory management	35
RES-16	Product strategies will be more precise, reducing the stocking of less needed assortments. It will entail changes in the procurement processes—rethinking and re-operationalizing.	Assortment rationalization	15
		Reconfiguration of purchasing processes	30
	More distribution centres will be created and logistics hubs will reappear at regional level.	Decentralization of distribution centres	34
	Warehousing conditions in particular will change—greater control of stock levels and the introduction of customer-specific security features.	Logistics processes reconfiguration	47
	For security reasons the frequency and size of deliveries will change—purchases will be more frequent and smaller.	Increasing the frequency of deliveries	12
RES-17	Our company has decided to increase the hourly rate for employees in the US, Canada and some European countries. We have also hired additional staff to handle e-commerce orders, allowing flexible working hours to match staffing capacity.	Increasing the salaries of employees	19
		Changing the forms of employment to flexible ones	21
	We have provided new contactless delivery methods and special conditions for the collection of purchases for disabled customers and those aged 60 +.	Reconfiguration of customer order fulfilment process	41
		Developing e-commerce distribution	39
	We have lifted the restrictions on the quantity of products sent to the picking centres.	Increasing inventory of finished products on SC demand side	14
	We have significantly developed and expanded our online sales platform to reach customers who have so far had no contact with online shopping.	Developing relationships with customers using online platforms	40
RES-18	Sales targets are set for purchases, so to save our results it was decided to use domestic suppliers.	Increasing the importance of local suppliers in isolated regions	24
RES-19	The company now faces a significant challenge as 75% of its strategic product purchases came from Asia. Nevertheless, the company needs to focus on reducing stock outs. Deliveries must become closer and more frequent to ultimately ensure business continuity and avoid capital freezes. The company will bet on developing business relationships with domestic suppliers and those who could deliver relatively quickly (Europe).	Increasing the importance of local suppliers in isolated regions	24
RES-20	We base our production largely on ethanol and isopropyl alcohol. At the very beginning of the pandemic, this raw material almost disappeared from the market. The situation was improved by the start of production by the state-owned companies.	Domestic production	33
	Well-run relationships, mutual cooperation, flexibility on both sides—they build mutual trust and determine to whom the supplier will sell its products, at a time like this—that is, when they have to ration and choose who gets the goods. It seems to me that in the situation I have described, where access to raw materials is extremely difficult, the winners are those who have “done their homework” on supplier management, exchange of know-how and are able to maintain relations with suppliers online.	Know-how exchange between partners in the SC	22
		Developing relationships with customers using online platforms	40
RES-21	In the current situation, an exponential increase in orders in online shops can be observed. In the early stages of the outbreak, online sales platforms reported an increase in orders of between 150% and 240%.	Developing e-commerce distribution	39
	The huge downturn caused by the COVID pandemic will certainly prompt companies to diversify their suppliers. By concentrating all production in China and other Asian countries, we have faced, for example, a crisis in access to medicines (production of all ingredients and substances needed for the pharmaceutical industry is carried out in China) and the automotive and computer industries were also threatened by the downturn (deliveries of prefabricated and semi-finished products, etc., stopped at Chinese ports).	Searching for sources of supply for new purchase categories	25
	A pandemic may significantly reduce the level of globalization. Companies will look for suppliers from other markets, so the importance of local suppliers may increase. Purchasing strategies may change, as may the rules of contract.	Supplier relocation	31
RES-22	Companies that invest in new technologies benefit in the event of disruption. They are able to make an efficient analysis of how a particular phenomenon may affect their supply chain in the near term. When companies have knowledge of where the disruption will come from and which products will be affected, they have time to immediately implement avoidance and mitigation strategies by, for example, buying or controlling inventory allocation.	Checking the level of inventory in SC intermediary links	13
	Companies dependent on global sourcing face difficult crisis management choices in the wake of supply chain disruptions. A good solution is to monitor global suppliers. New technologies like artificial intelligence allow for extensive supplier monitoring.	Supplier base mapping and monitoring	7
RES-23	The criteria for selecting a supplier have changed—we do not focus primarily on price, but the following are important: financial situation of the company/sources of financing; assurance of continuity of supply and on-time delivery; flexible logistical minimum; payment terms.	Changing the criteria for selecting suppliers	1
RES-24	The company should also be aware of what the supplier's capacity is and to what extent it is affected by pandemics. By collecting and sharing this information internally, the purchasing function is able to respond more effectively to demand, as both it and other departments can be more aware of supplier capacity and any obstacles to order fulfilment resulting from a pandemic. What's more, mapping also allows more effective monitoring of the costs associated with working with a supplier, but also the costs associated with a pandemic: those caused by a lack of supply, factory closures or the need to deploy new suppliers.	Supplier base mapping and monitoring	7
RES-25	The drastic change in market conditions has forced us to temporarily reduce the salaries of our employees and management. At the same time, we looked for other ways to reduce operating costs. Production losses, which were previously acceptable, were reduced by rationalizing the product range.	Lowering the salaries of employees	20
		Changes in the product offer oriented to waste reduction	16

## Data Availability

The authors declare that all data supporting the findings of this study are available within the article and its supplementary information files.
