# Correction: Creating supply chain resilience during and post-COVID-19 outbreak: the organizational ambidexterity perspective

**DOI:** 10.1007/s40622-022-00322-z

**Published:** 2022-09-07

**Authors:** Barbara Ocicka, Wioletta Mierzejewska, Jakub Brzeziński

**Affiliations:** 1 Institute of Corporate Finance and Investment, SGH Warsaw School of Economics, al. Niepodległości 162, 02-554 Warsaw, Poland; 2 Institute of Management, SGH Warsaw School of Economics, al. Niepodległości 162, 02-554 Warsaw, Poland; 3 Department of Logistics, Faculty of Management, University of Lodz, ul. Matejki 22/26, 90-237, Łódź, Poland

## Correction to: DECISION (2022) 49:129–151 10.1007/s40622-022-00309-w

Unfortunately, in the original article the figures are wrongly published. The corrected version are updated in the original version.

